# Prenatal alcohol exposure perturbs the development of radial glial cells in the fetal olfactory bulb

**DOI:** 10.1111/acer.70161

**Published:** 2025-09-10

**Authors:** Yuka Imamura Kawasawa, Kazue Hashimoto‐Torii, Masaaki Torii, Fumiaki Imamura

**Affiliations:** ^1^ Department of Neuroscience and Experimental Therapeutics Penn State College of Medicine Hershey Pennsylvania USA; ^2^ Center for Neuroscience Research, Children's Research Institute Children's National Hospital Washington DC USA; ^3^ Department of Pediatrics, Pharmacology and Physiology, School of Medicine and Health Sciences George Washington University Washington DC USA

**Keywords:** development, fetal alcohol spectrum disorder, olfactory bulb, prenatal alcohol exposure, radial glial cell

## Abstract

**Background:**

Prenatal alcohol exposure (PAE) causes fetal alcohol spectrum disorder (FASD) and is associated with various cognitive and sensory impairments, including olfactory dysfunction. While both genetic and environmental factors contribute to olfactory dysfunction, PAE is considered a significant factor affecting brain development, including the olfactory system. In this study, we investigated the impact of PAE on the developing olfactory bulb (OB), specifically focusing on OB RGCs—radial glial cells that give rise to OB projection neurons.

**Methods:**

Ethanol was administered to pregnant mice at embryonic day (E) 11, a time point when OB RGCs generate the highest number of mitral cells—a major class of OB projection neurons. To investigate the impact of PAE on OB RGCs, BrdU was injected 30 min prior to ethanol administration to label OB RGCs in the S phase of the cell cycle. The location and differentiation of BrdU^+^ cells were subsequently examined in the developing OB at E11, E13, and E15. We also assessed whether inhibition of GABA(A) receptors could mitigate the effects induced by PAE.

**Results:**

PAE was found to impair the progression of migration of OB RGC nuclei to the apical side of the ventricular zone for mitosis, indicating the inhibition of the transition from the S phase to the M phase (G2/M arrest). Therefore, PAE delays neurogenesis of OB RGCs. Importantly, our findings suggest that GABAergic signaling mediated by the mTOR signaling plays a critical role in these PAE‐induced effects.

**Conclusions:**

These findings provide insights into the mechanisms by which PAE disrupts OB development by impairing neurogenesis of RGC, contributing to a better understanding of the underlying mechanisms of olfactory dysfunction observed in FASD.

## Graphical abstract

Prenatal alcohol exposure affects the development of radial glial cells (RGCs) in the embryonic mouse olfactory bulb. Ethanol administered at embryonic day 11, a time point when RGCs generate the largest number of mitral cells, caused G2/M arrest in RGCs, delaying their progression to mitosis and subsequent neuronal differentiation. This study also found that GABA(A) receptor‐mediated mTOR signaling downregulation contributes to these effects. These findings highlight a potential mechanism underlying the olfactory dysfunction observed in fetal alcohol spectrum disorder.

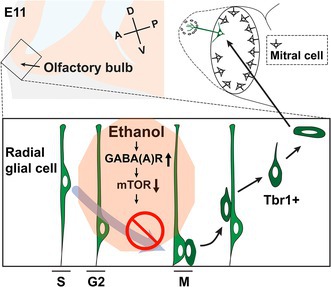

## INTRODUCTION

Fetal alcohol spectrum disorder (FASD) is a series of physiological and cognitive defects caused by prenatal alcohol exposure (PAE) (Popova et al., 2023). Alcohol exposure during fetal development disrupts neural development, and children with FASD frequently exhibit cognitive impairments and sensory deficits, including olfactory dysfunction associated with atypical eating habits (Amos‐Kroohs et al., 2016; Jirikowic et al., 2020). Furthermore, individuals with FASD often experience secondary conditions such as attention‐deficit/hyperactivity disorder (ADHD), depression, and anxiety (Hellemans et al., 2008; Mattson et al., 2011).

Olfaction, the sense of smell, is essential for survival, enabling animals to detect predators, locate prey, and assess environmental hazards. In humans, olfaction significantly influences mood, cognition, and overall quality of life. However, olfactory dysfunction, such as a decreased sense of smell (hyposmia) or the complete loss of smell (anosmia), affects approximately 2%–25% of the general population and can significantly impact their quality of life (Lee et al., 2013; Yang & Pinto, 2016). Individuals with olfactory dysfunction may experience challenges in food selection, social interactions, and even an increased risk of depression and anxiety disorders (Kohli et al., 2016; Lee et al., 2013). This impairment can arise from various factors, including chronic sinusitis, aging, and neurodegenerative diseases such as Parkinson's and Alzheimer's diseases.

Beyond acquired causes, olfactory dysfunction can also be congenital. Approximately one in 10,000 individuals are born with olfactory disorders (Doty, 1995), often associated with genetic conditions such as Kallmann syndrome and CHARGE syndrome (Deller et al., 2022). However, a significant proportion of congenital cases have no identifiable genetic cause and are considered idiopathic (Kamarck et al., 2024). Notably, people born with olfactory dysfunctions experience higher social insecurity and are at increased risk of depression than those who have normal olfaction (Croy et al., 2012; Lemogne et al., 2015). PAE is another significant contributor to olfactory dysfunction (Akers et al., 2011; Imamura, 2024), and the interplay between olfactory dysfunction and psychiatric disorders in FASD warrants further investigation.

To elucidate the mechanisms underlying olfactory dysfunction in FASD, this study aimed to investigate the effects of PAE on the development of the olfactory system. Given that many individuals with congenital olfactory disorders exhibit abnormalities in the development of the olfactory bulb (OB) (Koenigkam‐Santos et al., 2011; Pinto et al., 2005), a critical brain structure for olfactory processing, and that OB is one of the most vulnerable brain structures to PAE (Akers et al., 2011), we focused on OB development. Particularly, we investigated the effects of PAE on the generation of mitral cells, major projection neurons of the OB, from radial glial cells in the developing OB (OB RGCs). Previous studies have demonstrated a strong correlation between abnormalities in mitral cell development and olfactory dysfunction (Bastakis et al., 2015; Cai et al., 2023; Li et al., 2019). Recognizing that alcohol‐induced damage to neural stem cells plays a critical role in neurodevelopmental abnormalities (Adams et al., 2023; Delatour et al., 2019; Di Rocco et al., 2019; Lu et al., 2023; Sambo et al., 2022; Wilhelm & Guizzetti, 2015), this study investigated the impact of PAE on stem cells that produce mitral cells—OB radial glial cells (OB RGCs). Our findings demonstrate that PAE significantly impairs the cell cycle and neurogenesis of OB RGCs.

## MATERIALS AND METHODS

### Animals

CD1 mice (Charles River; Wilmington, MA; strain code 022; RRID: IMSR_CRL:22) were used for all the experiments in this study. The day on which we found a copulation plug was determined as embryonic day (E) 0, and the succeeding days of gestation were numbered consecutively. All protocols were approved by the Institutional Animal Care and Use Committee (IACUC) of Penn State College of Medicine, and all methods were performed by following their guidelines.

### Prenatal administration of BrdU, ethanol, and bicuculine

5‐bromo‐2′‐deoxyuridine (BrdU; Sigma) was intraperitoneally injected into pregnant mothers at E11 (50 mg/kg). Injections were performed between 10 am and noon. Fetuses were exposed to ethanol via oral gavage or intraperitoneal injection (i.p.) of ethanol to the mothers 30 minutes after the BrdU administration.

For oral gavage, pregnant mothers were anesthetized with isoflurane, and 25% ethanol in PBS (3.0 g/kg) was administered by intragastric gavage using a 24 G animal feeding needle. For i.p., pregnant mice received intraperitoneal injections of 25% ethanol in PBS (4.0 g/kg) using a 27 G needle. When ethanol is administered intragastrically (3.0 g/kg) and intraperitoneally (4.0 g/kg) to mice, blood alcohol concentration (BAC) reaches 150–200 mg/dL and 300–350 mg/dL in 30 min, respectively, mimicking intoxication (Carson & Pruett, 1996; Livy et al., 2003; Pruett et al., 2020). PBS alone was used as the control. To inhibit the GABA(A) receptor, the stock solution of bicuculine (10 mg/mL in DMSO) was diluted with PBS and intraperitoneally injected into the pregnant mothers (1 mg/kg) 10 min after the ethanol administration.

Prenatal embryos at E11 (90 min after ethanol/PBS administration), E13, or E15 were harvested and fixed in 4% paraformaldehyde overnight after pregnant mothers were euthanized with CO_2_ inhalation. The fixed embryos were cryopreserved in 30% sucrose (wt/vol) in PBS, embedded in an optimal cutting temperature compound, and kept at −80°C until used for immunohistochemistry.

### Immunohistochemistry

The olfactory tissues were cut on a cryostat into 20‐*μ*m slices and stored at −80°C until use. The slices were pretreated for 30 min in 0.025 M HCl at 65°C and rinsed with 0.1 M borate buffer (pH 8.5), PBS, and TBS‐T (10 mM Tris–HCl (pH 7.4), 100 mM NaCl with 0.3% Triton X‐100 (vol/vol)). The slices were then blocked with blocking buffer (5% normal donkey serum (vol/vol) in TBS‐T) at 20–25°C for 1 h and incubated with primary antibodies diluted in blocking buffer overnight at 4°C. Sections were washed with TBS‐T and then incubated with secondary antibodies with 4′6‐diamino‐2‐phenylindole dihydrochloride (DAPI; Thermo Fisher Scientific; #D1306 RRID:AB_2629482) for nucleus staining for 1 h. The immunoreacted sections were washed and coverslipped with a Fluoro‐Gel mounting medium (Electron Microscopy Science).

The primary antibodies used were rat anti‐BrdU (1:200; Accurate Chemical & Scientific Corp; OBT0030, RRID:AB_609568), mouse anti‐BrdU (1:50; DSHB; #G3G4; RRID:AB_2314035), rabbit anti‐Tbr1 (1:5000; Abcam; #ab31940, RRID:AB_2200219), mouse antineural cell adhesion molecule (NCAM) (1:500; Sigma‐Aldrich; #C9672, RRID:AB_1079450), and rabbit antiphospho‐mTOR (1:100; Cell Signaling Technologies; #5536; RRID:AB_10691552).

The secondary antibodies used were donkey antimouse IgG Cy2 (1:200; Jackson ImmunoResearch Labs; Cat# 715‐225‐151, RRID:AB_2340827), goat antimouse IgG1 Alexa Fluor 488 (1:300; Thermo Fisher Scientific; #A‐21121, RRID:AB_2535764), donkey antirat Alexa Fluor 488 (1:300; Thermo Fisher Scientific; #A‐21208, RRID:AB_141709), and donkey antirabbit Alexa Fluor 555 (1:300; Thermo Fisher Scientific; #A‐31572, RRID:AB_162543).

### Imaging and data analysis

Images were captured with a Zeiss Axio Imager.M2 microscope with a Zeiss ApoTome.2 (Carl Zeiss AG). Levels were adjusted using the Photoshop software (Adobe; RRID: SCR_014199) but the images were otherwise unaltered. To quantify the distribution of BrdU^+^ cells in the developing OB, the ventricular zone of the presumptive OB (pOB) was divided into six sublayers from deep to superficial, and the percentages of BrdU^+^ cells in each sublayer were calculated. To quantify the percentages of BrdU^+^ cells expressing Tbr1, cells were manually counted (*n* = 3 animals per condition). For each condition and time point, three animals from one or two mothers were used for quantification, and results were presented as mean ± standard deviations. The results were statistically analyzed using a t‐test or one‐way ANOVA followed by the Tukey's HSD test.

## RESULTS

**FIGURE 1:**
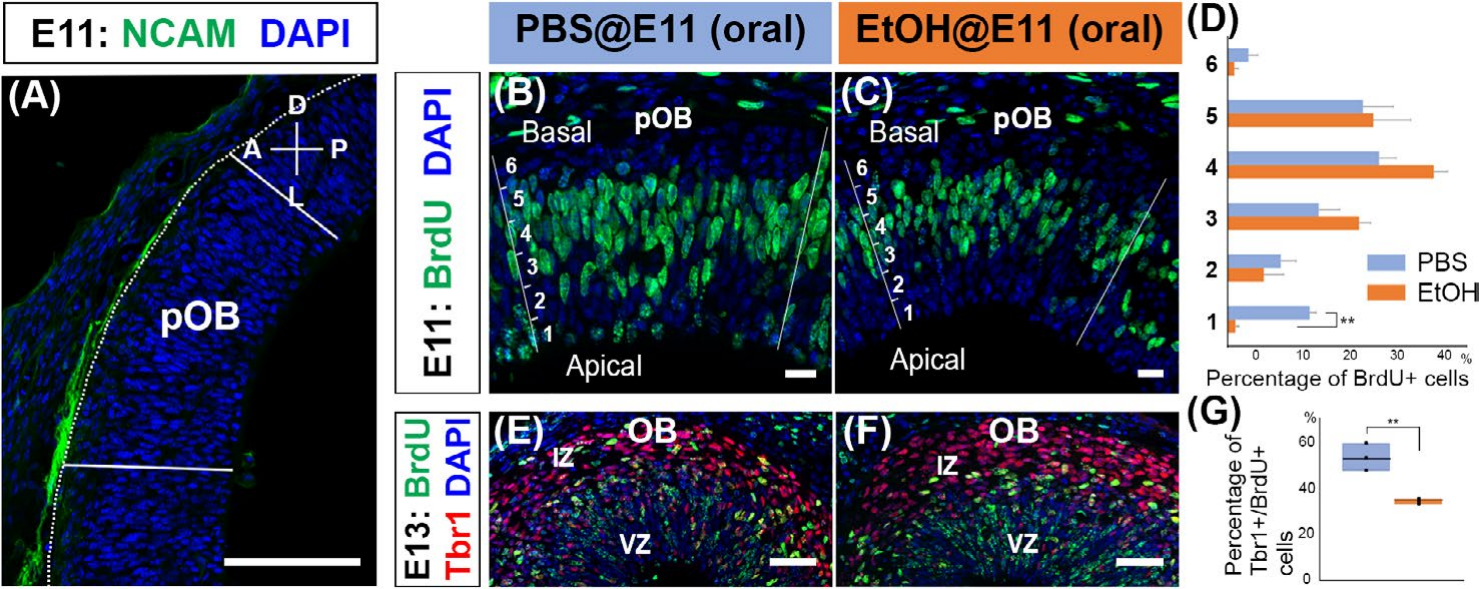
Defects in OB RGC development caused by oral administration of ethanol. (A) The sagittal section of the E11 mouse brain was immunostained with an antibody against NCAM (green), which is expressed by axons of olfactory sensory neurons. A region where NCAM^+^ olfactory axons target is defined as presumptive OB (pOB). (B–D) Pregnant female mice were injected with BrdU at E11 and 30 min later administered PBS (B) or ethanol (3 g/kg; C) by oral gavage. The embryos were fixed 90 min after the administration of PBS or ethanol, and the brain sections including pOB were stained with anti‐BrdU antibody (green). The pOB was defined based on the NCAM^+^ staining in adjacent sections and divided into six sublayers from deep to superficial, and the percentages of BrdU^+^ cells in each sublayer were calculated and quantified using a Student's *t*‐test (**p < 0.01; *n* = 3 animals from 1 and 2 mothers for PBS and ethanol, respectively) (D). (E–G) Embryos exposed to BrdU and PBS (E) or ethanol (F) at E11 were fixed at E13. OBs were stained with anti‐BrdU (green) and anti‐Tbr1 (red) antibodies. Percentages of BrdU^+^ cells expressing Tbr1 in the OB were calculated and quantified using a Student's *t*‐test (**p < 0.01) (*n* = 3 animals from 1 mother for both PBS and ethanol) (G). All nuclei were stained with DAPI (blue). Scale bars: 100 *μ*m in (A), 20 *μ*m in (B, C), and 50 *μ*m in (E, F).

Mitral cells originate from OB RGCs within the presumptive OB (pOB) (Imamura & Greer, 2013). Postmitotic cells derived from OB RGCs between embryonic days (E) 9 and 13 differentiate into mitral cells, with peak generation occurring around E11 (Blanchart et al., 2006; Imamura et al., 2011). To investigate the effects of PAE on OB RGC proliferation and differentiation into mitral cells, pregnant mice at E11 were intraperitoneally injected with BrdU, followed by oral administration of ethanol (25%; 3 g/kg) 30 min later. Ninety minutes postalcohol administration, the embryos were euthanized and processed for immunohistochemistry to detect cells labeled with BrdU in the pOB. Due to the difficulty in morphologically defining the pOB at this early developmental stage, we defined the pOB based on the projection of NCAM‐positive olfactory sensory neuron axons (Figure 1A) (Imamura & Greer, 2013). Localization of BrdU‐labeled cells in the pOB was compared between the control group treated with PBS and the experimental group exposed to ethanol. Previous studies have shown that during the S phase, radial glial nuclei are typically located on the basal side of the ventricular zone (VZ), while during the M phase (mitosis), they migrate to the apical ventricular surface to divide (Laguesse et al., 2015). Since BrdU is incorporated into DNA, a significant number of BrdU‐labeled RGC nuclei, which were in the S phase at the time of BrdU injection, are expected to be found in the apical region 120 min after BrdU injection. In the control group, many BrdU‐labeled nuclei were observed in the deep region (apical side) of the VZ. In contrast, the reduced number of BrdU‐labeled nuclei migrated to the apical VZ in the ethanol‐exposed group (Figure 1B–D). These findings suggest that PAE impairs the normal cell cycle progression of RGCs, potentially affecting the transition from the S phase to the M phase and/or the interkinetic nuclear migration toward the apical surface.

Next, we examined the differentiation of OB RGCs into mitral cells. After the asymmetric division in the apical VZ, some postmitotic cells lose the feature of RGCs, migrate toward the intermediate zone (IZ), and start to sequentially express Tbr1 and Tbr2 (Imamura & Greer, 2013). Specifically, Tbr1 is a transcription factor essential for the generation of glutamatergic neurons, and Tbr1 mutant mice lack mitral cells in the OB and show impaired olfactory discrimination (Bulfone et al., 1998; Huang et al., 2019). To investigate the impact of PAE on this differentiation process, we examined Tbr1 expression in BrdU‐positive cells at E13, 2 days after BrdU and ethanol (or PBS) administration. Because the cell counts of BrdU^+^ and Tbr1^+^ cells can vary significantly between animals and across OB slices, we calculated the percentage of BrdU^+^ cells that express Tbr1. In the control OB administered with PBS, many BrdU‐labeled cells were located in the IZ, and approximately 53% of them expressed Tbr1 (Figure 1E,G), which is consistent with our previous report (Imamura & Greer, 2013). However, in the OBs derived from ethanol‐exposed embryos, only about 34% of BrdU‐labeled cells were Tbr1^+^, and the Tbr1‐negative BrdU‐labeled cells were predominantly located in the VZ (Figure 1F,G). We also observed Tbr1^+^ cells that were not labeled with BrdU in the IZ. Mitral cells are predominantly generated between E10 and E12, with a peak at E11 (Imamura et al., 2011). We therefore assume that these Tbr1^+^/BrdU^−^ cells mostly represent postmitotic cells that were generated by OB RGCs prior to BrdU administration at E11. These findings suggest that PAE affects the generation of glutamatergic neurons from OB RGCs, potentially delaying the genesis of postmitotic cells that differentiate into glutamatergic neurons or diverting their fate away from mitral cells.

**FIGURE 2:**
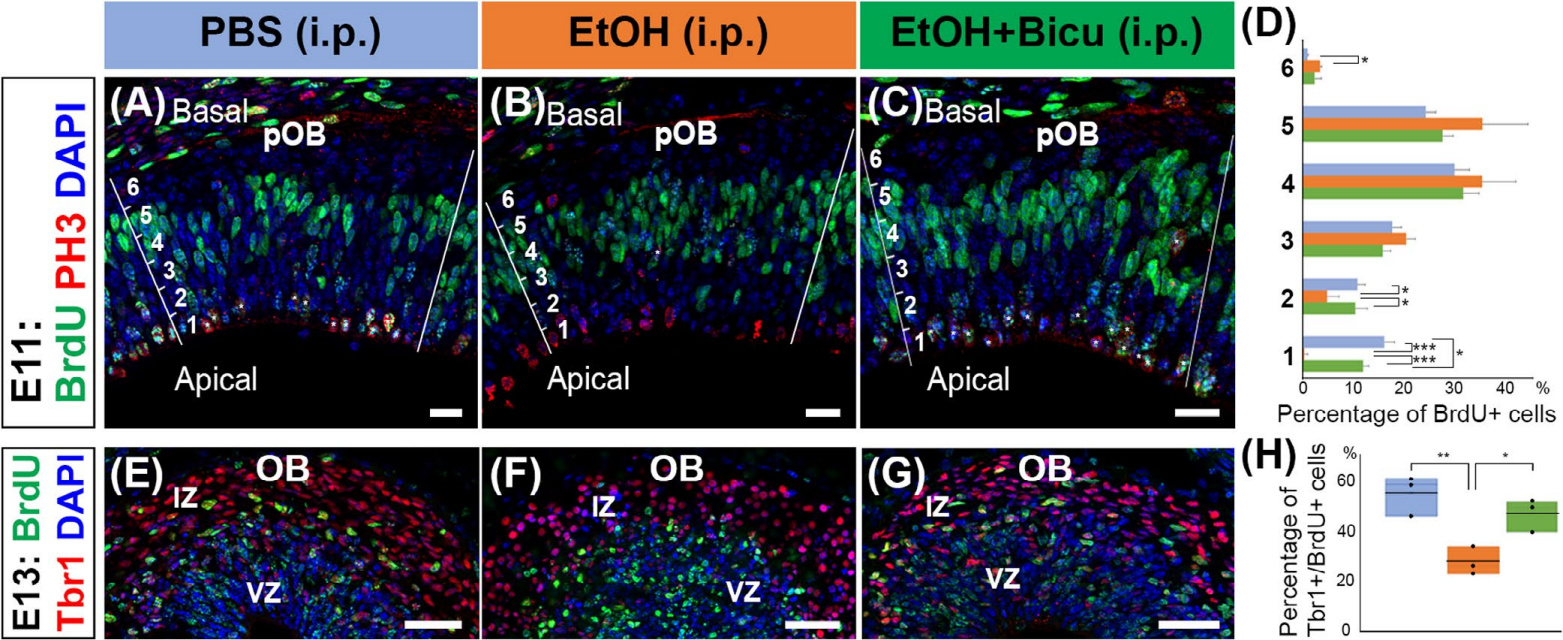
Defects in OB RGC development caused by intraperitoneal administration of ethanol. (A–D) Pregnant female mice were injected with BrdU at E11 and 30 min later intraperitoneally administered PBS (A) or ethanol (4 g/kg; B). Bicuculline (1 mg/kg) was administered 10 min after the ethanol administration (C). The embryos were fixed 90 min after the administration of ethanol or PBS, and the brain sections including pOB were stained with anti‐BrdU antibody (green) and anti‐PH3 antibody (red). The pOB was defined based on the NCAM^+^ staining in adjacent sections and divided into six sublayers from deep to superficial, and the percentages of BrdU+ cells in each sublayer were calculated and quantified using one‐way ANOVA followed by Tukey HSD test (*p < 0.05; **p < 0.01; *n* = 3 animals from 1, 1, and 2 mothers for PBS, ethanol, and ethanol + bicuculline, respectively) (D). Cells that are double‐positive with BrdU and PH3 in the pOB are marked with asterisks. (E–H) Embryos exposed to BrdU and PBS (E), ethanol (F), or ethanol + bicuculline at E11 were fixed at E13. OBs were stained with anti‐BrdU (green) and anti‐Tbr1 (red) antibodies. Percentages of BrdU^+^ cells expressing Tbr1 in the OB were calculated and quantified using one‐way ANOVA followed by Tukey HSD test (*p < 0.05; **p < 0.0; *n* = 3 animals from 2 mothers for all PBS, ethanol, and ethanol + bicuculline) (H). All nuclei were stained with DAPI (blue). Scale bars: 20 *μ*m in (A–C) and 50 *μ*m in (E–G).

In addition to oral administration, intraperitoneal ethanol administration to pregnant mice has been commonly used to model PAE. While this route bypasses the gastrointestinal tract and thus differs from the actual route of exposure via alcohol consumption, it is less invasive, can accurately control the dose, pattern, timing, and achievement of high blood alcohol concentration (BAC), and, therefore, is widely used to study the effect of acute PAE on brain development (Hwang et al., 2024; Mews et al., 2019; Mohammad et al., 2020; Mooney & Varlinskaya, 2011; Sambo et al., 2022). To confirm that the effects of ethanol on OB RGCs are independent of the exposure route, we also administered ethanol intraperitoneally to pregnant mice (25%, 4 g/kg) at E11, 30 min after the BrdU administration. Our results showed that the intraperitoneal ethanol administration to pregnant mothers disrupted the nuclear migration of OB RGCs toward the apical surface while no significant defect was observed in the saline‐injection control group (Figure 2A,B,D). To further investigate the cell cycle progression in OB RGCs, we examined the expression of phospho‐histone H3 (PH3) in BrdU+ nuclei 2 h after BrdU injection (i.e., 90 min after PBS/ethanol administration). PH3+ cells, which mark cells in M phase, were localized at the apical surface of the VZ. In the control pOB, many PH3+ cells were colabeled with BrdU, indicating normal progression from the S phase to M phase. In contrast, the ethanol‐exposed pOB showed a marked reduction in BrdU+/PH3+ cells, suggesting that PAE disrupts the transition from the S phase to the M phase, possibly causing a G2/M arrest in OB RGCs (Figure 2A,B). Moreover, the intraperitoneal administration of ethanol reduced the proportion of BrdU‐labeled cells expressing Tbr1 in E13 OB to about 27% (Figure 2E,F,H). These findings suggest that intraperitoneal ethanol administration significantly impairs the development of OB RGCs similar to oral administration, adequately reproducing the effects of maternal alcohol consumption on the fetus.

Since ethanol enhances the effects of GABA by allosterically modulating GABA(A) receptors (Förstera et al., 2016; Kumar et al., 2009), we further investigated the role of GABA(A) receptors in ethanol‐mediated effects on OB RGC cell cycle and neurogenesis. Pregnant mice at E11 were intraperitoneally injected with BrdU (50 mg/kg), followed by ethanol (4 g/kg) 30 min later, and then bicuculline (1 mg/kg), a GABA(A) receptor antagonist, 10 min after ethanol administration. Brains were collected 80 minutes postinjection of bicuculline (120 and 90 min after BrdU and ethanol injection, respectively) and analyzed for the localization of BrdU‐labeled cells in VZ. As a result, in the group administered bicuculline after ethanol exposure, more BrdU‐labeled cells coexpressing PH3 were localized at the apical VZ compared to the nonbicuculline group, and their distribution was similar to that of the nonethanol‐exposed control group (Figure 2C,D). Furthermore, in brains collected 2 days after ethanol exposure (E13), the proportion of BrdU‐labeled cells expressing Tbr1 in the OB was significantly higher in the bicuculline‐treated group compared to the ethanol‐only group and recovered to the level of the control group (Figure 2G,H). These findings suggest that ethanol‐induced G2/M arrest of OB RGCs and impaired Tbr1 expression in postmitotic cells are mediated by overactivation of GABA(A) receptors.

**FIGURE 3:**
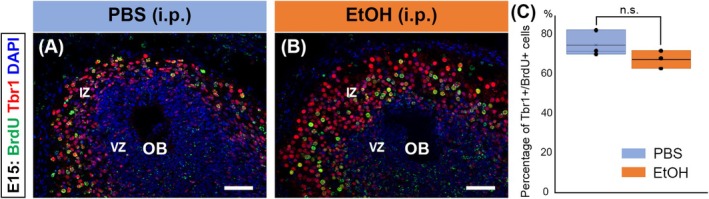
No significant PAE‐induced defect in the fate determination of OB RGC. (A–C) Pregnant female mice were injected with BrdU at E11, and 30 min later, intraperitoneally administered PBS (A) or ethanol (4 g/kg; B) were fixed at E15. OBs were stained with anti‐BrdU (green) and anti‐Tbr1 (red) antibodies. Percentages of BrdU^+^ cells expressing Tbr1 in the OB were calculated and quantified using a Student's *t*‐test (*n* = 3 animals from 2 and 1 mothers for PBS and ethanol, respectively) (HC). No significant difference was observed between the OBs treated with PBS and ethanol. All nuclei were stained with DAPI (blue). Scale bars: 50 *μ*m.

Our results suggest that PAE potentially delays the genesis of postmitotic cells or diverts their fate away from mitral cells. To examine the fate of OB RGCs labeled with BrdU, we analyzed the expression of Tbr1 in BrdU‐labeled nuclei at E15, 4 days after BrdU administration. In the PBS‐exposed control OB, about 75% of BrdU‐labeled cells were Tbr1^+^, which is consistent with our previous report (Imamura & Greer, 2013). Interestingly, about 68% of BrdU‐labeled cells expressed Tbr1 in the ethanol‐exposed group, which is comparable to PBS‐exposed groups (Figure 3A–C). This result indicates that PAE at E11 delays the neurogenesis of OB RGCs into mitral cells but does not change the fate of postmitotic cells.

**FIGURE 4:**
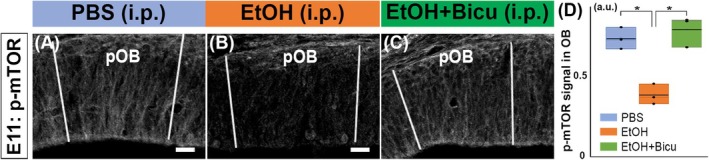
Downregulation of mTOR signaling caused by PAE. (A–D) Pregnant female mice were injected with BrdU at E11, and 30 min later, they were intraperitoneally administered PBS (A) or ethanol (4 g/kg; B). Bicuculline (1 mg/kg) was administered 10 min after the ethanol administration (C). The embryos were fixed 90 min after the administration of ethanol or PBS, and the brain sections, including pOB, were stained with an anti‐phospho‐mTOR (p‐mTOR) antibody. The pOB was defined based on the NCAM^+^ staining in adjacent sections. Ethanol exposure causes downregulation of phospho‐mTOR (p‐mTOR) (A, B), which is rescued by following bicuculline administration (C). Phospho‐mTOR level is shown with an arbitrary unit (a.u.) calculated based on the expression of mTOR in the skin, where the intensity did not change with ethanol (D). The values were quantified using one‐way ANOVA followed by the Tukey HSD test (*p < 0.05; *n* = 3 animals from 1, 1, and 2 mothers for PBS, ethanol, and ethanol + bicuculline, respectively). Scale bars: 50 *μ*m.

We further investigated the molecular mechanisms underlying the effects of PAE on OB RGCs. Previous studies suggest that PAE alters mTOR (mammalian target of rapamycin) signaling in the developing brain (Salem et al., 2021). This finding was also supported by our immunohistochemical examination, which revealed decreased levels of phosphorylated mTOR (p‐mTOR) in the pOB after ethanol exposure (Figure 4B,C,E). Importantly, bicuculline administration following ethanol injection rescued the downregulation of p‐mTOR (Figure 4D,E). These results suggest that GABAergic signaling mediated by the mTOR signaling plays a critical role in PAE‐induced impairment of OB RGCs.

## DISCUSSION

This study aimed to elucidate the effects of PAE on brain development, particularly the OB. Our findings revealed that PAE at E11 disrupted the cell cycle of OB RGCs and delayed their genesis of postmitotic cells that differentiate into mitral cells, providing significant insights into the underlying mechanisms of the developmental defect of OB observed in FASD.

A previous single‐cell RNA‐seq study showed that PAE (exposed to ethanol vapor for 45 min at E12.5) altered neural maturation and the cell cycle of cells examined 2 days after PAE (E14.5) (Salem et al., 2021). This study also suggested that PAE caused significantly differential expression of the genes enriched in pathways related to eukaryotic initiation factor (eIF2) and mTOR. Our study also demonstrated that PAE causes the downregulation of mTOR signaling in OB RGCs, which also aligns with previous research demonstrating that alcohol exposure during pregnancy alters the activation of molecules involved in mTOR signaling in the fetal brain (Lee et al., 2020). Notably, conditional knockout of mTOR in nestin+ neural stem cells suppressed the proliferation of neural progenitors, resulting in a decreased number of intermediate neural progenitors and a smaller brain size (Ka et al., 2014). Therefore, disruption of mTOR signaling in RGCs may be a critical cause of developmental defects of the brain in FASD children.

How does the downregulation of mTOR affect the development of RGCs? It is suggested that dysregulation of mTOR signaling disrupts the morphology, migration, and mitotic behavior of human outer RGCs (Andrews et al., 2020). This study demonstrated that PAE inhibits the transition of OB RGCs from the S‐phase to the M‐phase (G2/M arrest). A similar phenomenon has also been observed in the fibroblastic cell line (Mikami et al., 1997). It was shown that the activity of mTORC1 oscillates from highest in S/G2 to lowest in M/G1 and promotes the transition into the M phase (Joshi et al., 2024; Odle et al., 2020). Downregulation of mTOR signaling in the S/G2 phase might prevent the OB RGCs from entering the M‐phase. Furthermore, mTOR signaling regulates the activity of Rho GTPases, such as CDC42, and cyclin‐dependent kinases to control actin cytoskeletal dynamics and cell cycle progression in outer RGCs (Andrews et al., 2020; Joshi et al., 2024; Odle et al., 2020). Future studies include the investigation of the involvement of these molecules in ethanol‐induced G2/M arrest of OB RGCs. Nevertheless, the cell cycle is regulated by a complex network of signaling pathways. Therefore, ethanol may affect other signaling pathways besides mTOR, such as the p53, MAPK, and PI3K‐Akt pathways, which are interconnected with mTOR and involved in cell proliferation and survival (Panwar et al., 2023). Additionally, although our study focused on the G2/M arrest, mTOR signaling is also crucial for regulating cell cycle progression during the G1 and S phases (Proud, 2010). Further studies are needed to examine the effects of ethanol on RGCs during these phases of the cell cycle.

In addition to the defects in OB RGC development caused by PAE, G2/M arrest and neurogenesis delay, this study also reports that these defects could be rescued by inhibition of the GABA(A) receptor following ethanol exposure. In the fetal brain, the expression of the Na^+^/K^+^/Cl^−^ cotransporter (NKCC1) is high, while K^+^/Cl^−^ cotransporter (KCC2) expression is low, leading to elevated intracellular chloride concentrations (Schulte et al., 2018). It is said that, during development, GABA depolarizes RGCs to decrease DNA synthesis, inhibit cell cycle progression, and may regulate cell migration (Wang & Kriegstein, 2009). Since ethanol increases GABAergic neurotransmission by allosterically modulating the GABA(A) receptor (Förstera et al., 2016; Kumar et al., 2009), it can be speculated that ethanol enhances these mechanisms in OB RGCs and may cause malignant effects on their development. Rescue of mTOR activation by inhibition of GABA(A) receptor demonstrates that PAE‐induced downregulation of mTOR in OB RGCs is mediated by overactivation of GABA(A) receptors.

Our findings suggest that PAE delays the mitral cell genesis from OB RGCs but does not change the fate of postmitotic cells. Previous studies investigating the effects of alcohol on cultured stem cells and brain slices have reported not only delays in neurogenesis but also delays in cell migration, alterations in cell fate, and impairments in survival (Kumada et al., 2006; Nash et al., 2012; Taléns‐Visconti et al., 2011; Vallés et al., 1996; Vemuri & Chetty, 2005). A future study could investigate the effects of PAE on the total number and spatial distribution of mitral cells in the postnatal OB. In this study, the embryos were exposed to ethanol once at E11, which causes the temporal elevation of BAC (Livy et al., 2003). Repeated or chronic exposure to ethanol may lead to more persistent and severe consequences on OB RGC development (Imamura, 2024). Future studies should investigate the effects of PAE on OB RGCs by changing the timing, duration, and doses of alcohol exposure. The potential sex‐specific effects of alcohol and the role of epigenetic mechanisms in mediating the consequences of PAE should also be explored (Hwang et al., 2024; Mooney & Varlinskaya, 2011; Ungerer et al., 2013). Furthermore, since OB RGCs undergo direct neurogenesis to differentiate into mitral cells, whereas cortical RGCs generate pyramidal neurons through intermediate neural progenitors (Cardenas et al., 2018; Hevner, 2019; Imamura & Greer, 2013), it is important to clarify the similarities and differences in the effects of PAE between OB RGCs and cortical RGCs. Revealing the genes and signaling pathways susceptible to PAE in developing OB will provide new molecular targets for the treatment and prevention of olfactory dysfunction and associated psychological disorders in FASD children.

## CONFLICT OF INTEREST STATEMENT

The authors declare that the research was conducted in the absence of any commercial or financial relationships that could be construed as a potential conflict of interest.

## FUNDING INFORMATION

The PA Tobacco Settlement Fund; The Children's Miracle Network.

## Data Availability

The data that support the findings of this study are available from the corresponding author upon reasonable request.
